# Hepatitis C Virus NS3/4A Inhibition and Host Immunomodulation by Tannins from *Terminalia chebula*: A Structural Perspective

**DOI:** 10.3390/molecules27031076

**Published:** 2022-02-05

**Authors:** Vishal S. Patil, Darasaguppe R. Harish, Umashankar Vetrivel, Subarna Roy, Sanjay H. Deshpande, Harsha V. Hegde

**Affiliations:** 1 ICMR-National Institute of Traditional Medicine, Nehru Nagar, Belagavi 590010, India; vishalpatil6377@gmail.com (V.S.P.); vumashankar@gmail.com (U.V.); sanjay.deshpande2389@gmail.com (S.H.D.); harshah@icmr.gov.in (H.V.H.); 2 ICMR-National Institute for Research in Tuberculosis, Chetpet, Chennai 600031, India; 3 Regional Centre for Biotechnology, NCR-Biotech Science Cluster, Faridabad 121001, India

**Keywords:** 1,2,3,4,6-Pentagalloyl glucose, chebulagic acid, docking, dynamics, hepatitis C virus NS3/4A, network pharmacology, *Terminalia chebula* Retz

## Abstract

*Terminalia chebula* Retz. forms a key component of traditional folk medicine and is also reported to possess antihepatitis C virus (HCV) and immunomodulatory activities. However, information on the intermolecular interactions of phytochemicals from this plant with HCV and human proteins are yet to be established. Thus, by this current study, we investigated the HCV NS3/4A inhibitory and host immune-modulatory activity of phytocompounds from *T. chebula* through in silico strategies involving network pharmacology and structural bioinformatics techniques. To start with, the phytochemical dataset of *T. chebula* was curated from biological databases and the published literature. Further, the target ability of the phytocompounds was predicted using BindingDB for both HCV NS3/4A and other probable host targets involved in the immune system. Further, the identified targets were docked to the phytochemical dataset using AutoDock Vina executed through the POAP pipeline. The resultant docked complexes with significant binding energy were subjected to 50 ns molecular dynamics (MD) simulation in order to infer the stability of complex formation. During network pharmacology analysis, the gene set pathway enrichment of host targets was performed using the STRING and Reactome pathway databases. Further, the biological network among compounds, proteins, and pathways was constructed using Cytoscape 3.6.1. Furthermore, the druglikeness, side effects, and toxicity of the phytocompounds were also predicted using the MolSoft, ADVERpred, and PreADMET methods, respectively. Out of 41 selected compounds, 10 were predicted to target HCV NS3/4A and also to possess druglike and nontoxic properties. Among these 10 molecules, Chebulagic acid and 1,2,3,4,6-Pentagalloyl glucose exhibited potent HCV NS3/4A inhibitory activity, as these scored a lowest binding energy (BE) of −8.6 kcal/mol and −7.7 kcal/mol with 11 and 20 intermolecular interactions with active site residues, respectively. These findings are highly comparable with Asunaprevir (known inhibitor of HCV NS3/4A), which scored a BE of −7.4 kcal/mol with 20 key intermolecular interactions. MD studies also strongly suggest that chebulagic acid and 1,2,3,4,6-Pentagalloyl glucose as promising leads, as these molecules showed stable binding during 50 ns of production run. Further, the gene set enrichment and network analysis of 18 protein targets prioritized 10 compounds and were predicted to potentially modulate the host immune system, hemostasis, cytokine levels, interleukins signaling pathways, and platelet aggregation. On overall analysis, this present study predicts that tannins from *T. chebula* have a potential HCV NS3/4A inhibitory and host immune-modulatory activity. However, further experimental studies are required to confirm the efficacies.

## 1. Introduction

It is well known that hepatitis C viral infection caused by the hepatitis C virus (HCV) is a major worldwide health problem associated with serious liver disease [1,2]. Chronic infection by HCV includes liver fibrosis, cirrhosis, and hepatocellular carcinoma (HCC) that eventually lead to liver failure. HCV affects 2–3% of the population, ~200 million people worldwide [3,4]. The current gold standard of treatment for HCV infection includes subcutaneous injection of PEG-IFN-α2b or PEG-IFN-α2a plus oral administration of Ribavirin [5,6]. This treatment regimen showed an eradication of the infection in 75–90% of patients with HCV genotypes 2 or 3 and 40–50% of patients with HCV genotypes 1 or 4. However, in many clinical trials, ~10–15% of the patients discontinued this treatment due to the onset of side effects viz. fatigue, anemia, thrombocytopenia, neutropenia, moderate to severe depression, ophthalmologic disorders, headache, fever, myalgia, and so on [7,8,9,10,11,12,13,14]. Hence, the identification of potential hits with better safety and efficacy against HCV and its associated complications has become a global emergency.

**Figure 1:**
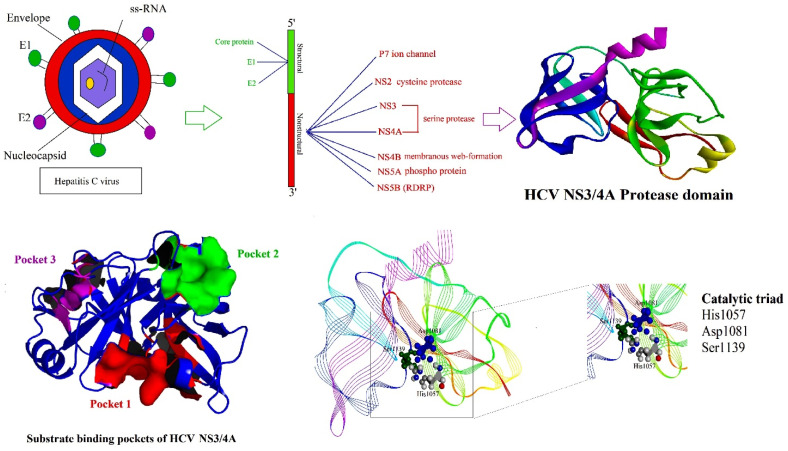
HCV, NS3 X-ray crystallographic structure, substrate binding pockets, and its catalytic triad residues.

HCV is a small enveloped positive-sense single-stranded RNA virus that belongs to the *Hepacivirus* genus of the family Flaviviridae [15]. The HCV NS3/4A serine protease is a key protein consisting of a catalytic subunit (the N-terminal one-third of the NS3 protein) and an activating cofactor (NS4A protein) that hydrolyses four known sites along the viral polyprotein, thus produce nonstructural (NS) proteins which are essential for viral maturation, RNA replication, and hence viral replication [16,17,18,19]. Importantly, the proteolytic cleavage of HCV polyprotein into nonstructural (NS) proteins, i.e., NS4A, 4B, 5A, and 5B is mainly catalyzed by NS3/4A protease. The catalytic triad of HCV NS3 contains three essential residues viz., His1057, Asp1081, and Ser1139, which are involved in the acid–base catalysis on target peptides and are strictly conserved among all HCV-derived sequences [20]. Ser1139 executes a nucleophilic attack on the carbonyl moiety of the substrate and His1057 allows the catalysis to progress, while Asp1081 aids in the stabilization of His1057 by hydrogen bonding and thereby increasing its pKa value [21]. Figure 1 represents the proteins encoded by HCV genome organization, X-ray crystallographic structure of NS3/4A (substrate binding pockets and catalytic sites).

The current anti-HCV therapy includes the long-term utilization of antiviral drugs that interact with the NS3/4A substrate binding pockets, but these binding regions have evolved with several common mutations viz., Q1080K/R, R1155K/Q, A1156T, and D1168A/V/T/H, which could ultimately cause drug resistance, alter the mode of action, and cause adverse drug reactions [22]. In the present study, an attempt has been made to identify the novel bioactive molecules having nontoxic effects and that could act on subpocket active site residues, i.e., catalytic triad residues to decrease mutation-related drug resistance and adverse-drug reactions (ADRs).

HCV, in 50–80% of infected individuals, causes cirrhosis and HCC when the immune system is compromised due to hepatic damage, inflammatory milieu, and when it fails to fight the virus [2]. During the viral life cycle, the NS3 protein of HCV serves as a viral protease, an RNA helicase, and a nucleoside triphosphatase (NTPase) and stimulates hepatic fibrosis, which accelerates the progression of liver disease [23]. HCV NS3/4A proteolytic activity antagonizes various host adaptor molecules, ultimately affecting the innate immunity via immune intracellular signaling pathways, which play a critical role in the failure of viral clearance and promote liver inflammation [24,25]. HCV NS3/4 binds to p53 (a tumor suppressor), forms a complex, inhibits its function, and ultimately contributes to HCC progression [26]. Previous studies reported that, in mitochondria, HCV NS3/4A targets and cleaves the IPS-1 adaptor protein molecule to inhibit IFN α/β mediated immune defenses [25,27], and the NS3/4A protease complex also disrupts the retinoic acid-inducible gene I (RIGI) and toll-IL-1 receptor domain-containing adaptor inducing IFN-β (TRIF) thereby triggering nuclear factor-kappa β (NFkβ) and IFN regulatory factor (IRF)-3 [28], which are essential for innate immunity. Hence, the HCV NS3/4A protease represents a potential target for the identification of novel anti-HCV hits that can interact with the active site (catalytic triad) of NS3/4A and are also involved in the modulation of the immune system to suppress chronic HCV infection.

Medicinal plants and their products have been extensively utilized against various viral infections and their associated complications. A complex mixture of phytocompounds is known to suppress the progression of viral infection via inhibiting viral replication and by acting as an immune modulator via targeting viral particles, multiprotein molecules, and multipathways associated with viral pathogenesis [29]. *Terminalia chebula* Retz. (Haritaki) is a member of the Combretaceae family, known as a “King of Medicine or Arura”, because in Tibetan medicine it is considered to be a great panacea due to its beneficial effects on all diseases caused by Pitta, Vayu, and Kapha. It is also shown to cure diseases of all seven Dhatus viz., plasma, blood, muscle, fat, bone, marrow/nerve, and reproductive tissue [30,31,32]. *T. chebula* is rich in tannins (pyrogallol category) like chebulagic acid, ellagic acid, corilagin, punicalagin, neochebulinic acid, chebulanin, chebulinic acid, 3,4,6-trio-glloyl-D-glucose, 1,6-dio-galloyl-D-glucose, casuarinin, terchebulin, and 1,2,3,4,6-penta-O-galloyl-β-D-glucose, etc. It also contains phenolic compounds, viz., galloylglucose, corilagin, terflavin A, punicalagin, triterpene maslinic acid, and so on [33]. This plant is known to possess broad-spectrum activities, viz., antiviral, antiprotozoal, antibacterial, antifungal, etc., and has anticancer [34], antidiabetic [35], hepatoprotective [36,37], immunomodulatory [38], anti-inflammatory [30], wound healing [39,40], cardio-protective [41,42], anti-caries [43], etc., activities. Hydrolyzable tannins from *T. chebula* are well documented for antiviral activities [44], viz., anti-HCV [45,46], anti-HSV-1 [47], anti-HSV-2 (HSV, herpes simplex virus) [48] activity via inhibiting entry and multiple viral targets.

**Figure 2:**
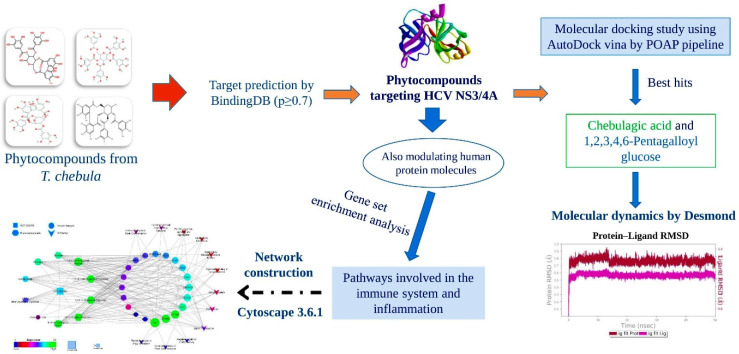
Workflow followed in the current study.

Hence, based on the antiviral, immunomodulatory, anti-inflammatory, and hepatoprotective reports of *T. chebula*, we framed the current study to identify the potential phytocompounds from *T. chebula* that could effectively target the active site of HCV NS3/4A and also potentially modulate the host immune system, through multiprotein and multipathway mechanisms. This analysis was performed by utilizing data from an experimentally based ligand–protein interaction database, gene set enrichment analysis, network pharmacology, molecular docking, and molecular dynamics simulation studies. The complete workflow of the current study is illustrated in Figure 2.

## 2. Materials and Methods

### 2.1. Retrieval of Phytocompounds

The structural details of reported phytochemicals from *T. chebula* were retrieved from the literature, phytochemical databases, viz., the Phytochemical Interaction database (https://www.genome.jp/db/pcidb/ Accessed on 2 September 2020), Dr. Duke’s database (https://phytochem.nal.usda.gov/ Accessed on 2 September 2020), and the Database of Ethnomedicinal Plants of Western Ghats [49]. The list of phytocompounds with their references is provided in Table S1 (Please see the supplementary material).

### 2.2. Target Identification

The canonical SMILES of the phytochemicals of *T. chebula* were retrieved from the PubChem chemical database (https://pubchem.ncbi.nlm.nih.gov/ Accessed on 2 September 2020) and were screened in BindingDB (https://www.bindingdb.org/bind/index.jsp Accessed on 4 September 2020) with a similarity score threshold (probability value) of ≥0.7. BindingDB currently contains over 20,000 ligand-protein interactions data for over 11,000 different small-molecule ligands and 110 different drug targets [50,51]. Thus, the gene ID of each predicted protein molecule was retrieved from the UniProt protein database (https://www.uniprot.org/ Accessed on 4 September 2020) and was further processed with a gene set enrichment analysis.

### 2.3. Gene Set Enrichment Analysis

The gene IDs of the identified protein targets were queried in the STRING 11.0v database (Search Tool for the Retrieval of Interacting Genes/Proteins (https://string–db.org) Accessed on 10 September 2020) and the enriched molecular pathways via analyzing the Reactome biological pathways (https://reactome.org Accessed on 10 September 2020) using a ≤0.07 false discovery rate (FDR) were retrieved. Further, the pathways associated with the progression of HCV infection and the immunomodulation of the host were segregated via comparing with the reported pathways available in the literature and the KEGG hepatitis C pathway (https://www.genome.jp/kegg-bin/show_pathway?hsa05160 Accessed on 10 September 2020). The reported pathways involved in the pathogenesis of HCV infection and immune system modulation are listed in Table S2 (Please see the supplementary material).

### 2.4. Network Construction

Cytoscape v3.6.1 software [52] was utilized to construct the phytocompound–protein-pathway network. The network was analyzed via a network analyzer by treating the network as a “treat network as a direct” command. The degree of interaction between phytocompound–protein pathways was analyzed by the topological parameter “edge count”. The edge count was applied to “low values to small sizes” for node size and “low values to bright colors” for node color. The network was designed by utilizing the layout “degree sorted circular layout” [53,54,55].

### 2.5. Molecular Docking Studies

#### 2.5.1. Preparation of Ligand and Protein

The structural coordinates of phytocompounds and Asunaprevir (a known inhibitor) were downloaded in .sdf format from PubChem (https://pubchem.ncbi.nlm.nih.gov/ Accessed on 15 September 2020). To avoid interference, clashes within ligand atoms, and to produce a reasonable staring pose during docking, each compound was energy minimized by applying the mmff94 force field using Open Babel. Further, the e poses having the lowest potential energy were selected and saved in .pdb format. Subsequently, Open Babel was also utilized to convert the .pdb format to the .pdbqt format. The 3D X-ray crystallographic structure of HCV NS3/4A (PDB ID: 4WF8) was retrieved from RCSB PDB (https://www.rcsb.org/ Accessed on 15 September 2020) and was refined by a two-step atomic-level energy minimization using ModRefiner (https://zhanglab.ccmb.med.umich.edu/ModRefiner/ Accessed on 15 September 2020). The PROCHECK Ramachandran plot was utilized to check the plausibility of the structure (https://servicesn.mbi.ucla.edu/PROCHECK Accessed on 15 September 2020). The overall quality factor was also checked by the ERRAT scoring (https://servicesn.mbi.ucla.edu/ERRAT/ Accessed on 15 September 2020).

#### 2.5.2. Assessment of Active Site Residues

The active site amino acid residues of HCV NS3/4A were assigned based on the crystal structure of HCV NS3/4A protease in a complex with Asunaprevir having a PDB ID: 4WF8 (1.70 Å resolution) expressed in the *Escherichia coli* BL21(DE3) expression system. In addition, the druggable sites of HCV NS3/4A were also predicted using GalaxySite (http://galaxy.seoklab.org/cgi-bin/submit.cgi?type=SITE Accessed on 15 September 2020).

#### 2.5.3. Protein–Ligand Docking

The binding affinity of phytocompounds with HCV NS3/4A was predicted using a GNU parallel-based pipeline that integrates Open Babel and AutoDock suite (POAP) [56]. To start with, the ligand molecules were converted from .pdb to .pdbqt format. Prior to the docking process, the crystal structure of HCV NS3/4A (PDB ID: 4WF8) was subjected to a 50 ns molecular dynamics simulation and the conformation with the lowest potential energy was sampled and was used for subsequent docking studies. The Vina exhaustiveness was set to 100, and the grid box dimensions were set in accordance to the hotspots reported (center: x = 41.77, y = 14.28, z = 21.03; size: x = 42.45, y = 23.46, z = 32.63; spacing 1 Å). After docking, the intermolecular interactions of protein-ligand complexes were visualized in Discovery Studio Visualizer 2019 (DSV 2019). The complexes having the lowest binding energy (BE) and maximum interactions with the active site residues were considered for the MD analysis.

### 2.6. Molecular Dynamics (MD) Simulation

In order to validate the stability of complex formation, the protein–ligand complexes were subjected to a 50 ns MD simulation using the Desmond package [57]. The simple point charge water model (SPC) was used to solvate the system in a cubical box having 10 Å × 10 Å × 10 Å periodic boundary conditions. Further, the system was neutralized by the addition of Na^+^/Cl^−^ counterions. The SHAKE algorithm was applied to restrain the geometry of water molecules, bond lengths, and bond angles of heavy atoms. To calculate the long-range interactions, the particle mesh Ewald method was utilized and the Lennard–Jones interactions cutoff value was set to 10.0 Å. Further, the system was minimized/relaxed using default parameters. Finally, for 50 ns of production run, a Nosé–Hoover chain thermostat with 1.0 ps relaxation time and the Martyana–Tobias–Klein barostat method with 2.0 ps relaxation time was applied, with a Coulombic short range cutoff radius set to 9.0 Å, wherein the temperature and pressure were set to 300 K and 1.01325 bar, respectively. The root-mean-square deviation (RMSD), root-mean-square fluctuation (RMSF), and radius of gyration (rGyr) were analyzed to know the residue-wise fluctuations. Further, the ligand–protein contacts were checked after 50 ns simulation, in order to infer the stable intermolecular contacts between the protein and ligand throughout the production run.

### 2.7. Druglikeness, Side Effects, and Toxicity of Phytocompounds

The druglike property of phytocompounds was predicted based on the Lipinski rule of five by the MolSoft online server (https://molsoft.com/mprop/ Accessed on 10 September 2020). The MolSoft server predicts a positive and negative druglikeness score using chemical fingerprints. In addition, the ADVERpred online server [58] was utilized to predict four major side effects of the phytocompounds, i.e., hepatotoxicity, nephrotoxicity, arrhythmia, and myocardial infarction, caused by the small molecules. The phytocompounds were considered to be toxic if the probable activity (Pa) was ≥0.5 and probable inactivity (Pi) was ≤0.5. The PreADMET online server was also utilized to predict mutagenicity, 2-year carcinogenicity bioassay in mouse and rat, hERG inhibition, Ames test in TA100_10RLI, TA100, TA1535_10RLI, and TA1435_NA strains.

## 3. Results

### 3.1. Retrieval of Phytocompounds and Target Identification

Forty-two compounds reported to be present in *T. chebula* were retrieved from the documented reports and phytocompounds databases (Table S1) and were subjected to target prediction using BindingDB, which inferred 190 probable protein targets (Table S3 in supplementary material). Among forty-two compounds, ten compounds were predicted to target HCV NS3/4A and all were identified as tannins (Table 1).

### 3.2. Gene Set Enrichment Analysis and Network Analysis

**Figure 3:**
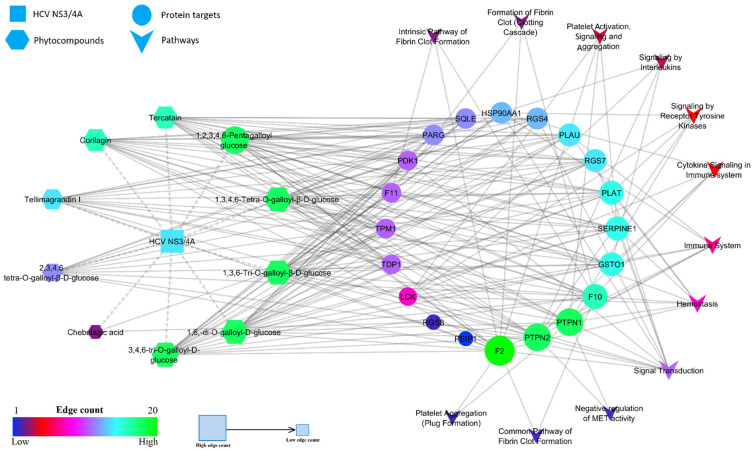
Network representation of phytocompound-target-pathway.

The other protein targets of HCV NS3/4A targeting phytocompounds were subjected to a gene set enrichment analysis. Among these, 10 compounds were predicted to target 18 protein targets (Table S4 in supplementary material). The enrichment analysis of these 18 targets revealed potential involvement in 19 molecular pathways (Table S5 in supplementary material). Among the 19 pathways, 9 pathways were found to be associated with HCV infection, liver fibrosis, cirrhosis, and HCC. F10, F11, F2, LCK, PLAT, PLAU, PTPN1, SERPINE1 were found to be involve in hemostasis and F2, GSTO1, HSP90AA1, LCK, PLAU, PTPN1, PTPN2 in the immune system. Moreover, 18 probable targets were also found to be involved in fibrin clot formation, platelet activation, signaling, and aggregation, interleukins, and tyrosine kinase signaling pathways (Table 2 and Figure 3).

### 3.3. Protein Quality Check and Stability Analysis by Molecular Dynamics Simulation

**Figure 4:**
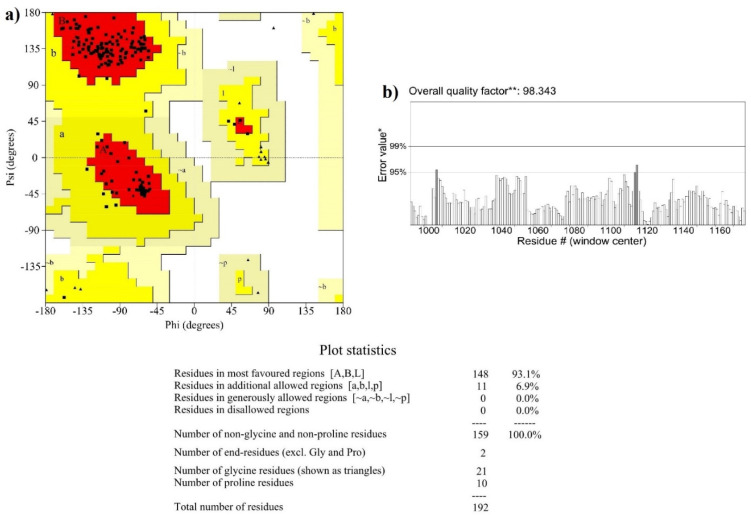
Characteristics of PDB ID: 4WF8 (HCV NS3/4A crystal structure). (**a**) Ramachandran plot by PROCHECK. (**b**) Overall quality prediction by ERRAT. * On the error axis, two lines are drawn to indicate the confidence with which it is possible to reject regions that exceed that error value. ** Expressed as the percentage of the protein for which the calculated error value falls below the 95% rejection limit.

**Figure 5:**
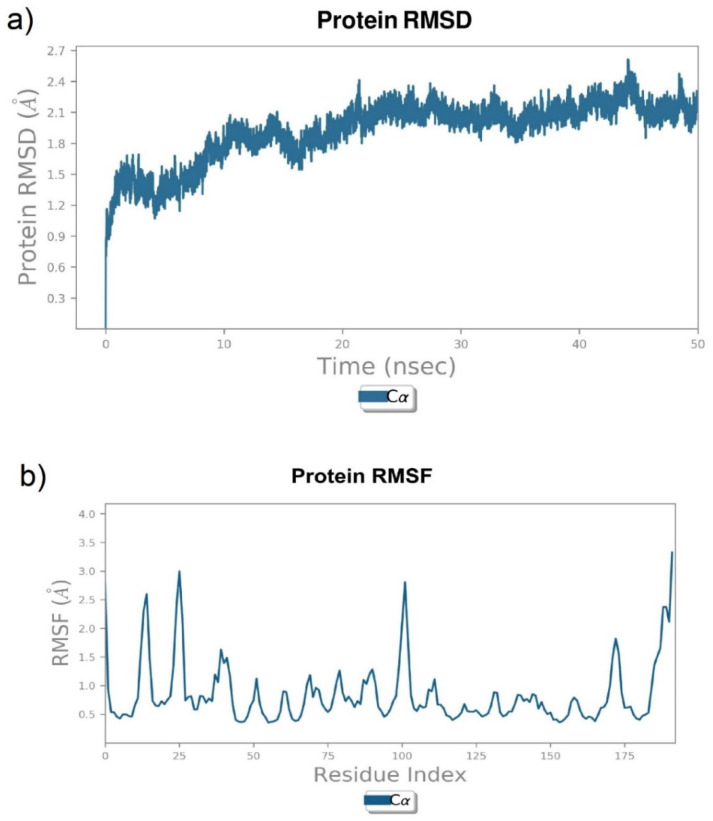
Stability of HCV NS3/4A (PDB ID: 4WF8). (**a**) Backbone RMSD and (**b**) per-residue backbone RMSF.

The Ramachandran plot of HCV NS3/4A was assessed by PROCHECK and the overall structural quality factor was scored by ERRAT. The number of residues in favored, additionally allowed, generously allowed, and disallowed regions were found to be 93.1, 6.9, 0, and 0%, respectively (Figure 4a). The overall quality of the protein was found to be 98.38% (Figure 4b). The protein molecule stability was assessed by a molecular dynamic simulation for 50 ns. Initially, higher RMSD fluctuations were observed between 0 to 10 ns ranging from 1.2 to 1.8 Å. After 10 ns, a stable RMSD trajectory was observed with fluctuations ranging from 1.8 to 2.1 Å (Figure 5).

### 3.4. Assessment of Active Site Residues

The active site residues as per PDB records were Ile994, Arg997, Tyr1006, Ala1007, Gln1008, Arg1011, Asp1025, Asn1027, Glu1030, Phe1043, His1057, Val1078, Asp1081, Cys1097, Cys1099, Arg1123, Ile1132, Leu1135, Lys1136, Gly1137, Cys1145, His1149, Phe1154, Arg1155, Ala1156, Ala1157, and Asp1168. The active site residues that were predicted from GalaxySite are Gln1041, Phe1043, His1057, Gly1058, Asp1081, Ile1132, Leu1135, Lys1136, Gly1137, Ser1138, Ser1139, Phe1154, Arg1155, Ala1156, Ala1157, Val1158V, and Asp1168. The amino acid residues Phe1043, His1057, His1057, Asp1081, Ile1132, Leu1135, Lys1136, Gly1137, Phe1154, Arg1155, Ala1156, Ala1157, and Asp1168 were found to be common among both predictions. Considering both predicted results and literature reports, the active cavity was defined for docking processes.

### 3.5. Ligand–Protein Docking

**Figure 6:**
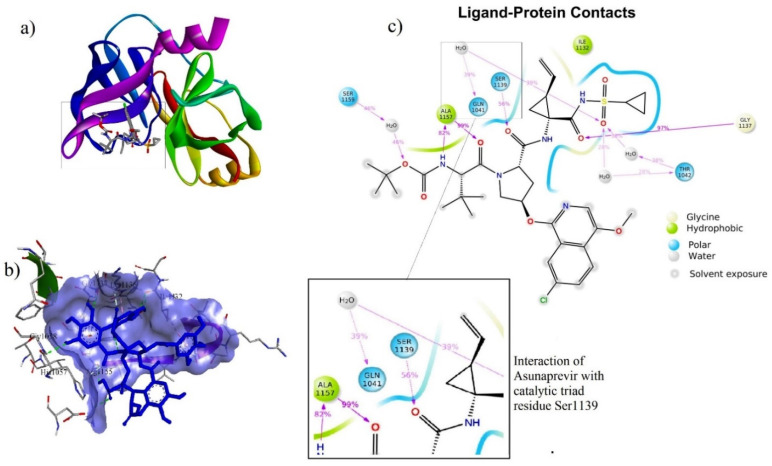
Intermolecular Interactions of Asunaprevir with NS3/4A. (**a**) Asunaprevir at catalytic triad residue site; (**b**) Asunaprevir at NS3/4A binding pocket; (**c**) Asunaprevir interaction with catalytic triad residue Ser1139.

**Figure 7:**
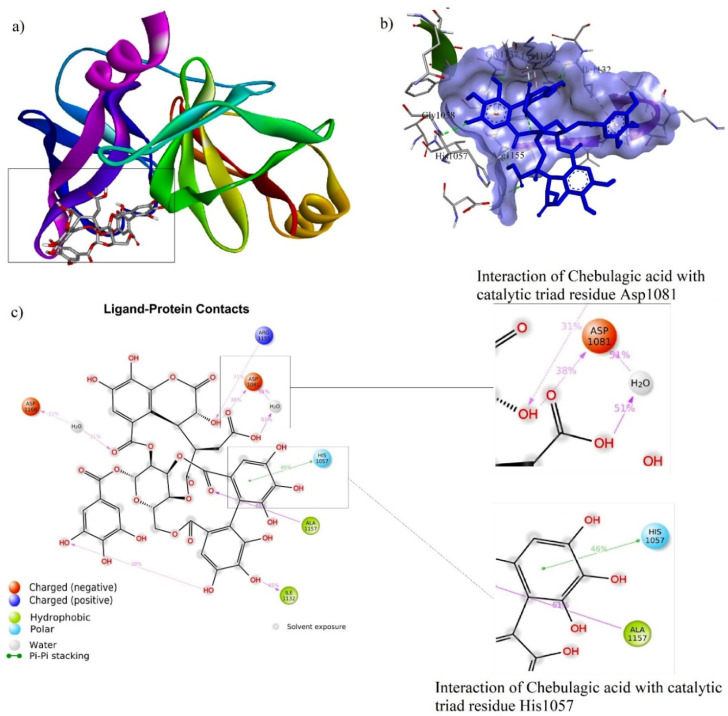
Intermolecular interactions of chebulagic acid with NS3/4A. (**a**) Chebulagic acid at catalytic triad residue site; (**b**) chebulagic acid bound to NS3/4A binding pocket; (**c**) chebulagic acid interactions with catalytic triad residue Ser1139, His1057, and Asp1081.

**Figure 8:**
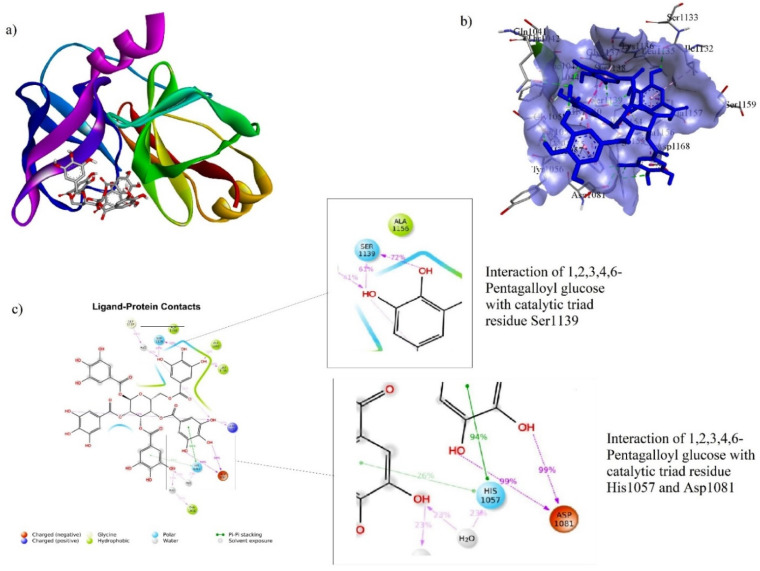
Intermolecular Interactions of 1,2,3,4,6-Pentagalloyl glucose with NS3/4A. (**a**) 1,2,3,4,6-Pentagalloyl glucose at catalytic triad residue site; (**b**) 1,2,3,4,6-Pentagalloyl glucose bound to NS3/4A binding pocket; (**c**) 1,2,3,4,6-Pentagalloyl glucose interactions with catalytic triad residue Ser1139, His1057, and Asp1081.

The binding energy (BE), hydrogen bond interaction (HBI), and non-hydrogen bond interaction (non-HBI) of phytocompounds and Asunaprevir with HCV NS3/4A are represented in Table 3. The standard molecule “Asunaprevir” scored the lowest BE of −7.4 kcal/mol via forming twenty intermolecular interactions with active site residues (four HBI, i.e., Gly1137…=O (2), Ala1157…NH, and Ala1157…=O and 16 non-HBI, i.e., Arg1155 (2), Asp1081 (2), His1057 (2), Ala1156 (4), Ile1132 (3), Val1158 (2), and Lys1136 (2)). Among the ten phytocompounds, chebulagic acid scored the lowest BE at −8.4kcal/mol via forming eleven intermolecular interactions with active site residues (eight HBI, i.e., Gly1058…O-, Ile1132…OH (2), Gly1137…OH, Arg1155…=O, Arg1155…OH, and Ser1159…OH (2), and four non-HBI, i.e., His1057, Ile1132, Lys1136, and Ser1139). Further, 1,2,3,4,6-Pentagalloyl glucose scored the second-lowest BE at −7.7 kcal/mol via forming twenty interactions with active site residues (ten HBI, i.e., Gln1041…OH, His1057…OH (2), Asp1081…OH, Ile1132…OH, Ser1139…O-, Ser1139…OH (2), and Arg1155…O- (2) and ten non-HBI, i.e., Val1055, His1057 (2), Gly1058, Ile1132, Lys1136, Ala1156 (3), and Ala1157). The intermolecular interactions of Asunaprevir, chebulagic acid, and 1,2,3,4,6-Pentagalloyl glucose with HCV NS3/4A are represented in Figure 6, Figure 7 and Figure 8, respectively.

### 3.6. Molecular Dynamics Simulation of NS3/4A–Ligand Complexes

The MD simulation of Asunaprevir, chebulagic acid and 1,2,3,4,6-Pentagalloyl glucose in a complex with HCV NS3/4A at 50 ns exhibited a very stable ligand RMSD (Å) for Lig fit Prot and showed the best fit of ligand on the protein RMSF (Å). The sustained ligand atom interactions with NS3/4A protein residues after a 50 ns MD simulation are shown in Table 4.

#### 3.6.1. Asunaprevir–NS3/4A Complex

**Figure 9:**
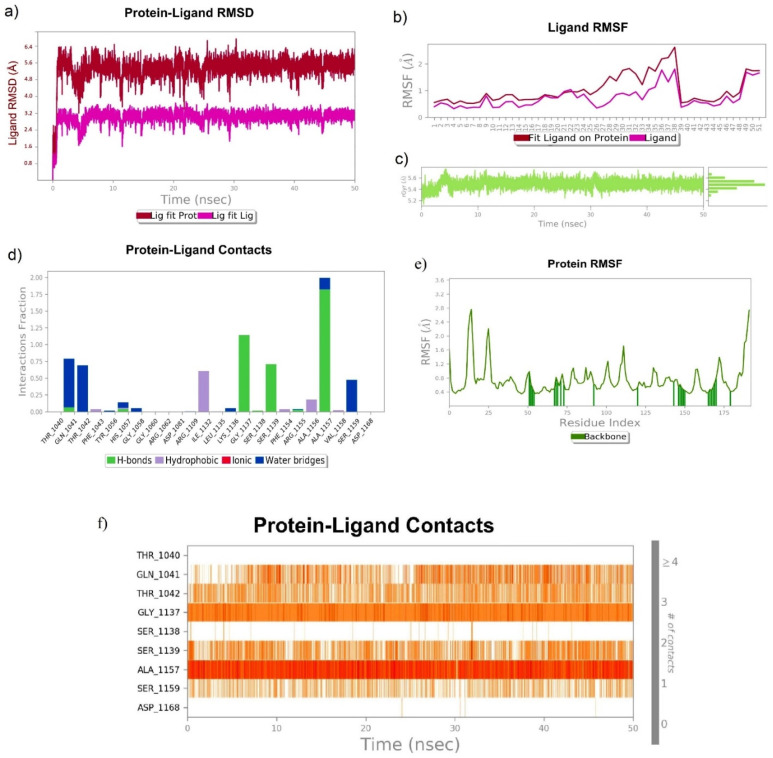
MD trajectories of Asunaprevir in complex with HCV NS3/4A for a 50 ns simulation: (**a**) RMSD, (**b**) RMSF, (**c**) rGyr, and (**d**–**f**) residue-wise ligand–protein contacts.

Asunaprevir in complex with NS3 4A showed a very stable ligand RMSD (Å) between 5.2 Å and 5.6 Å from 0 to 50 ns of simulation. Further, a slight fluctuation from 0 to 5 ns was observed for rGyr (Å) and found to get stabilized from 5 to 50 ns at 5.4 Å. Asunaprevir atom position 16 (=O), position 7 (=O), and position 44 (NH) formed very stable contacts with the residues Gly1137 (97%), Ala1157 (99%), Ala1157 (82%) of NS3/4A, respectively. Among the total interactions observed, Ala1157 and Gly1137 formed a very stable interaction throughout the simulation. However, a slight fluctuation between Asunaprevir and NS3/4A Gln1041, Thr1042, Ser1139 (catalytic triad residue), and Ser1159 was observed. Figure 9 represents the RMSD, RMSF, rGyr, and contacts of Asunaprevir and NS3/4A.

#### 3.6.2. Chebulagic Acid–NS3/4A Complex

**Figure 10:**
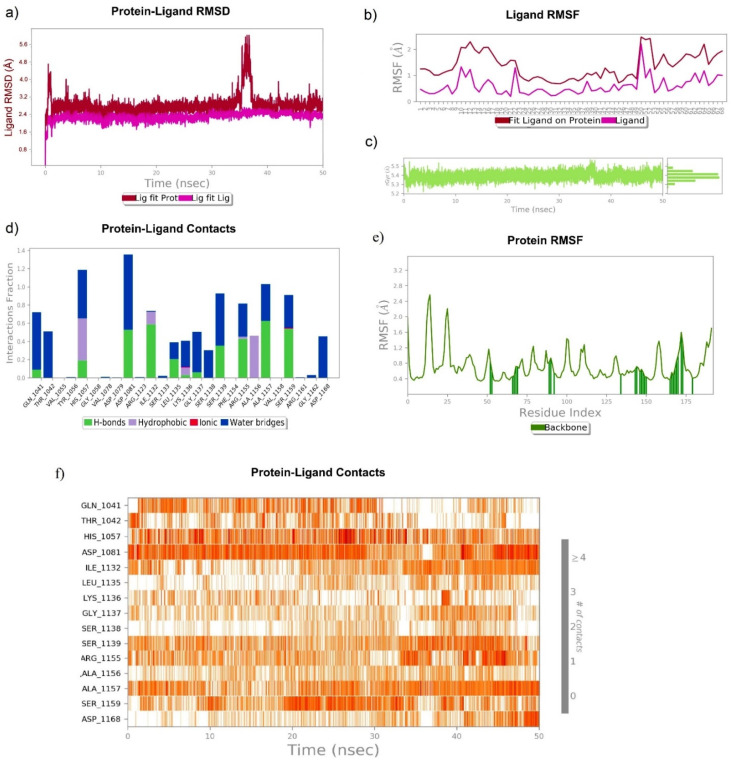
Stability of chebulagic acid with HCV NS3/4A at 50 ns of simulation: (**a**) RMSD, (**b**) RMSF, (**c**) rGyr, and (**d**–**f**) residue-wise ligand–protein contacts.

The chebulagic acid and NS3/4A complex exhibited a very stable ligand RMSD (Å) between 2.6 and 2.8 Å from 0 to 50 ns of simulation (Figure 10a), however it showed a fluctuation from 35 ns to 38 ns. Further, the complex showed the best fit on NS3/4A (Figure 10b). In addition, rGyr (Å) was found to be stable from 0 to 50 ns. Chebulagic acid atom position 41 (=O), position 68 (OH), and position 46 (OH) formed a very stable contact with the residues Ala1157 (61%), Asp1081 (51%), Ile1132 (45%) of NS3/4A, respectively, from 0 to 50 ns. Among the total interactions between chebulagic acid and NS3/4A, His1057 (catalytic triad residue) and Asp1081 (catalytic triad residue) showed very stable interactions throughout the simulation. However, slightly fluctuated interactions were observed between chebulagic acid and the Gln1041, Thr1042, Ile1132, Leu1135, Lys1136, Gly1137, Ser1138, Ser1139, Arg1155, Ala1156, Ala1157, Ser1159, and Asp1168 residues of NS3/4A.

##### 3.6.3. 1,2,3,4,6-Pentagalloyl Glucose–NS3/4A Complex

**Figure 11:**
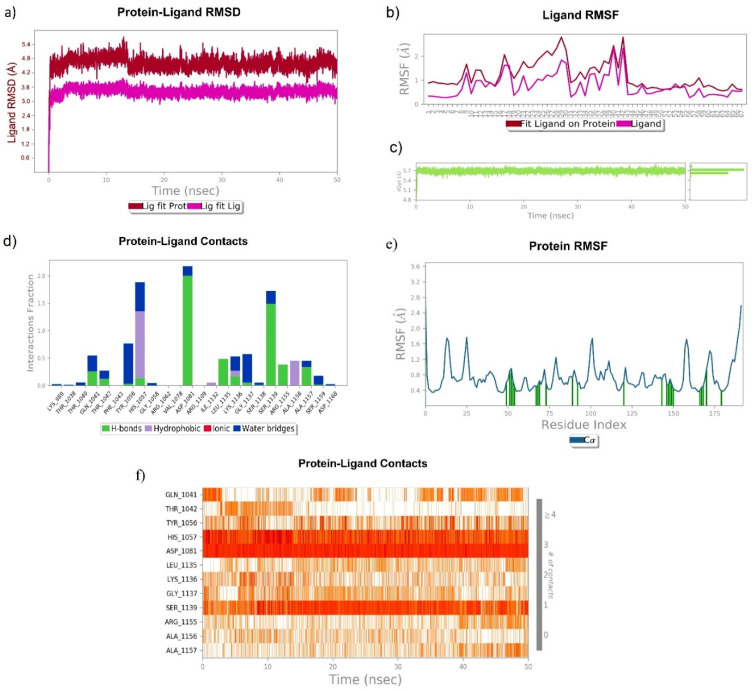
Stability of 1,2,3,4,6-Pentagalloyl glucose with HCV NS3/4A at 50 ns of simulation: (**a**) RMSD, (**b**) RMSF, (**c**) rGyr, and (**d**–**f**) residue-wise ligand–protein contacts.

1,2,3,4,6-Pentagalloyl glucose was also found to be the best hit for targeting NS3/4A, as it exhibited a very stable ligand RMSD (Å) between 4.4 and 4.8 Å from 0 to 50 ns (Figure 11a) and also showed the best fit on NS3/4A (Figure 11b). rGyr (Å) was found to be stable from 0 to 50 ns. 1,2,3,4,6-Pentagalloyl glucose atom position 40 (OH), 41 (OH), 65 (OH), 66 (OH) formed very stable contacts with the residues Asp1081 (99%), Asp1081 (99%), Ser1139 (61%), and Ser1139 (72%) of NS3/4A, respectively, from 0 to 50 ns. Among the total interactions observed, 1,2,3,4,6-Pentagalloyl glucose formed very stable interactions with His1057 (catalytic triad residue), Asp1081 (catalytic triad residue), and Ser1139 (catalytic triad residue). However, slightly fluctuated interactions were observed with the Gln1041, Thr1042, Tyr1056, Leu1135, Lys1136, Gly1137, Arg1155, Ala1156, and Ala1157 residues.

### 3.7. Druglikeness, Side Effects, and Toxicity of Phytocompounds

**Figure 12:**
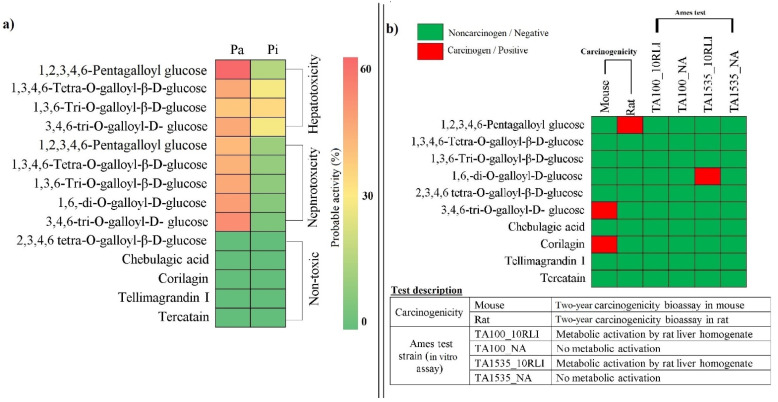
Heat map representation of phytocompounds with their probable (**a**) side effects and (**b**) toxicity.

The phytocompounds from *T. chebula* predicted to target HCV NS3/4A were further assessed for their druggability, side effects, and toxicity. Among the selected compounds, 1,3,4,6-Tetra-O-galloyl-β-D-glucose and 1,3,6-Tri-O-galloyl-β-D-glucose scored the highest druglikeness score (DLS) of 0.92 and 1,2,3,4,6-Pentagalloyl glucose scored the lowest 0.19, whereas chebulagic acid scored a DLS of 0.58. However, all the selected compounds were predicted to violate rule five due to the high molecular weight and ultimately high HBD and HBA. However, interestingly, all the compounds scored positive druglikeness scores. The druglikeness properties of phytocompounds are shown in Table 5. Among ten compounds, four compounds showed hepatotoxicity, five showed nephrotoxicity and five compounds were predicted to be nontoxic. However, the Pa value of five compounds having side effects was found to be ≤0.5, which indicated less probability to cause side effects (Table S6). Interestingly, chebulagic acid did not show any side effects. On looking at the carcinogenicity potential of 10 compounds, 1,2,3,4,6-Pentagalloyl glucose showed carcinogenicity in the rat, 3,4,6-tri-O-galloyl-D-glucose and corilagin showed carcinogenicity in the mouse. Only 1,6-di-O-galloyl-D-glucose showed the mutagenic property in TA1535_10RLI strain. All other selected compounds were found to be noncarcinogen and nonmutagen. The heat map in Figure 12a,b represents the side effects and toxicity profile of phytocompounds, respectively.

## 4. Discussion

In the present study, we investigated the HCV NS3/4A inhibitory and immune-modulatory activity of shortlisted phytocompounds from *T. chebula*. Ten phytocompounds from the *T. chebula* that are classified under the category of tannins were identified as a potent inhibitor of HCV NS3/4A via BindingDB and were also found to modulate the host immune system. Chebulagic acid and 1,2,3,4,6-Pentagalloyl glucose were identified as the best potentially active molecules against HCV NS3/4A as they scored the lowest BEs (−8.4 kcal/mol and −7.7 kcal/mol, respectively), and exhibited a maximum number of stable hydrogen bonded interactions with active site residues of HCV NS3/4A during the MD simulation. These molecules were also predicted to have optimal druggability and nontoxic effects. To date, numerous studies reported *T. chebula* as a potential anti-viral, viz., anti-HCV, anti-HBV, anti-HSV, etc., and a hepatoprotective herb [44,45,60,61]. Moreover, the compounds isolated from *T. chebula* were also reported to act on HCV targets [46], however, information on the intermolecular interactions of phytochemicals from this plant with HCV proteins and human targets are yet to be clearly established. The present study utilized information from BindingDB target prediction and chemoinformatics approaches to elucidate the anti-HCV and immune-modulatory effects of bioactive phytocompounds from *T. chebula*.

First, the shortlisted compounds were predicted for the probable inhibitory properties against HCV targets. As a result, ten compounds were found to target HCV NS3/4A with a probable score of ≥0.7. BindingDB compares the structure similarity of an unknown molecule with a known molecule and provides a *p*-value (similarity index/score) and a known compound IC_50_ value as outputs; based on this prediction the potential targets were prioritized. Duan et al. [46] reported the HCV NS3/4A inhibitory activity of Penta-O-galloyl-beta-D-glucoside (PubChem CID 15945060) by ELISA, and the IC_50_ value was found to be 0.75 µM. In the present study (Table 1), 1,3,4,6-Tetra-O-galloyl-β-D-glucose, 1,3,6-Tri-O-galloyl-β-D-glucose, 1,6,-di-O-galloyl-D-glucose, 2,3,4,6 tetra-O-galloyl-β-D-glucose, and 3,4,6-tri-O-galloyl-D-glucose from *T. chebula* scored the probable values of 0.98, 0.98, 0.94, 0.7, and 0.87, respectively, with reported IC_50_ of 0.75 µM for the known inhibitor, i.e., Duan et al. reported molecule Penta-O-galloyl-beta-D-glucoside to inhibit HCV NS3 protease. Similarly, chebulagic acid scored a *p*-value of 0.7 when compared with reported IC_50_ of 0.3 µM and 0.8 µM for compounds CID 511658 and 511659 from PubChem, respectively. Moreover, these compounds were reported to inhibit HCV NS3/4A in the HCV NS3 protease binding assay. Herein, we further aimed to infer the molecular interactions of these selected potential bioactive molecules with HCV NS3/4A via molecular docking and molecular dynamics simulation studies.

The docking study was carried for the standard HCV NS3/4A inhibitor Asunaprevir and selected phytocompounds against HCV NS3/4A by AutoDock Vina using a GNU parallel-based pipeline (POAP), as it enabled a high scalability, seamless operability, dynamic file handling, and optimal utilization of CPUs for computationally demanding tasks and it is also helpful in multireceptor docking [56]. Prior to the docking simulation, the X-ray crystallographic protein structure of NS3/4A (PDB ID: 4WF8) was loop-refined and validated for its structural stability using a 50 ns molecular dynamics simulation. The docking study results revealed that chebulagic acid had the highest binding affinity (−8.4 kcal/mol) with HCV NS3/4A and is found to interact with 11 key active site residues, whereas 1,2,3,4,6-Pentagalloyl glucose showed the highest (20) interactions with active site residues with the lowest BE of −7.7 kcal/mol. Importantly, all the predicted potential phytocompounds were found to establish stable intermolecular interactions with HCV NS3/4A active site residues. The known inhibitor Asunaprevir scored a BE of −7.4 kcal/mol with 20 interactions with active site residues. To validate the stability of complex formations Asunaprevir, chebulagic acid, and 1,2,3,4,6-Pentagalloyl glucose in complex with HCV NS3/4A, were subjected to an MD simulation of 50 ns. Asunaprevir, chebulagic acid, and 1,2,3,4,6-Pentagalloyl showed stable contacts with active site residues, as inferred through RMSD, RMSF, and rGyr trajectories.

Chebulagic acid is a benzopyran tannin isolated from *T. chebula* Retz., and a potent antiviral agent. Lin et al. [44] reported an antiviral activity of chebulagic acid in various viral cell lines, viz., hepatitis C virus (EC_50_ 12.16 μM in Huh-7.5 cells), human cytomegalovirus (EC_50_ 25.50 μM in HEL cells), dengue virus-2 (EC_50_ 13.11 μM in Vero cells), measles virus (EC_50_ 34.42 μM in CHO-SLAM cells), respiratory syncytial virus (EC_50_ 0.38 μM in Hep-2 cells), vesicular stomatitis virus (EC_50_ 61.28 μM in A549 cells), wild-type human adenovirus type-5 (EC_50_ 198.14 μM in A549 cells). Moreover, chebulagic acid was also shown to inhibit viral attachment, penetration, and spread [44]. Chebulagic acid is proven to confer inhibitory activity against HSV type 2 (HSV-2) and Enterovirus-71 with an IC_50_ of 31.84 µg/mL [48] and 12.5 μg/mL [62], respectively. Similarly, phytocompounds from *T. chebula* contains galloyl moiety, which is reported to possess anti-HCV activities. 1,2,6-tri-O-galloyl-b-D-glucose, 1,2,3,6-tetra-O-galloyl-b-D-glucose, and 1,2,3,4,6-penta-O-galloyl-b-D-glucose showed HCV NS3 protease inhibitory activity in ELISA with an IC_50_ of 1.89, 0.75, and 1.60 μM, respectively [46]. Moreover, Behrendt et al. [63] reported Pentagalloyl glucose as a highly bioavailable compound in mice that blocks HCV entry and improves antiviral efficacy of daclatasvir (a clinically used HCV inhibitor) and also found to inhibit Zika virus [63]. 1,2,3,4,6-Pentagalloyl glucose was also shown to confer anti-Rabies activity at an IC_50_ of 3.90 μM in baby hamster kidney-21 (BHK-21) cells [64].

Following the prioritization of HCV NS3/4A inhibitors from *T. chebula*, we further aimed the current study to infer the interaction probability of these inhibitors with human proteins and pathways by implementing gene set enrichment analysis using the Reactome database. The enriched interactions between compounds, human protein molecules, and pathways were constructed and analyzed through a network pharmacology approach. As a result, HCV NS3/4A inhibitors were also found to play a key role in the hemostasis, fibrin clot formation, signal transduction, platelet activation, aggregation, interleukins signaling, and the immune system via targeting LCK, PLAT, PLAU, SERPINE1, HSP90AA1, F10, F11, F2, PDK1, PTPN1, PTPN2, RGS4, RGS7, RGS8, and GSTO1 protein molecules. It is well known that the liver plays an important role in hemostasis, as it synthesizes multiple coagulation factors and proteins associated with fibrinolysis and produces thrombopoietin for platelet production. However, patients with chronic HCV infection and liver diseases are associated with lower levels of coagulation factors and thrombocytopenia, which contribute to an increased risk of bleeding, are directly associated with the immune response and immunomodulation and fail to combat the HCV, which ultimately leads to cirrhosis and hepatocellular carcinoma.

The utilization of currently available conventional drugs depends mainly on the concept of “single drug-single protein-single disease” that may not be sufficient in the treatment of infectious diseases [54]. Hence, the utilization of multicompounds belonging to the same drug class with smaller side effects and toxicity significantly increases the size of the druggability and exerts various pharmacological effects via network-dependent effects [65]. In the present study, all ten compounds showed positive DLS and were predicted to have nontoxic effects in mice, rats, and in various Ames test bacterial strains. Hence, the previous literature and the current study findings reflect the inhibition of HCV NS3/4A target and the regulation of multiple homeostatic proteins and pathways involved in the immune system in the management of HCV infection.

## 5. Conclusions

The present study employed target prediction using an experimentally determined drug–target interaction database, molecular pathways analysis by gene set enrichment and network pharmacology, compound–protein interactions by molecular docking, and molecular dynamics studies to identify the key ingredients from *T. chebula* with potential to inhibit HCV NS3/4A with immune-modulatory activity. Our study identified tannins from *T. chebula* to possess strong HCV NS3/4A inhibitory activity, in which chebulagic acid and 1,2,3,4,6-Pentagalloyl glucose were identified as most promising hits due to receptor specificity and minimal toxicity. Likewise, the gene set enrichment and network analysis identified *T. chebula* hydrolyzable tannins to play a major role in the immune system, homeostasis, signal transduction, cytokine signaling in the immune system, signaling by RTKs, interleukins, etc. The findings in this study strongly suggest the therapeutic potential of tannins from *T. chebula* as an anti-HCV and immune modulator in hosts. However, these findings are solely based on chemoinformatics approaches and thus demands further validation of chebulagic acid, 1,2,3,4,6-Pentagalloyl glucose or tannin-rich fraction in relevant cellular and in vivo models to corroborate the current findings.

## Figures and Tables

**Table 1:** List of phytocompounds from *T. chebula* predicted to inhibit HCV NS3/4A.

Phytocompounds	PubChem ID	Structural Similarity (*p* ≥ 0.7–1)	Experimental Assay	Reference Compound PubChem CID	Experimental IC_50_ of Reference Compound (µM)	References
1,2,3,4,6-Pentagalloyl glucose	374874	1	Inhibitory activity against HCV NS3 protease by ELISA	15945060	0.75	[46]
1,3,4,6-Tetra-O-galloyl-β-D-glucose	471531	0.98				
1,3,6-Tri-O-galloyl-β-D-glucose	452707	0.98				
1,6,-di-O-galloyl-D-glucose	91227631	0.94				
2,3,4,6 tetra-O-galloyl-β-D-glucose	101011018	0.7				
3,4,6-tri-O-galloyl-D-glucose	14188641	0.87				
Chebulagic acid	250397	0.7	HCV NS3 protease binding assay	511658	0.3	[59]
				511659	0.8	[59]
Corilagin	73568	0.74	HCV NS3 protease binding assay	511658	0.3	[59]
				511659	0.8	[59]
			Inhibitory activity against HCV NS3 protease by ELISA	15945060	0.75	[46]
Tellimagrandin I	442690	0.7	HCV NS3 protease binding assay	511658	0.3	[59]
				511659	0.8	[59]
			Inhibitory activity against HCV NS3 protease by ELISA	15945060	0.75	[46]
Tercatain	14411426	0.75	HCV NS3 protease binding assay	511658	0.3	[59]
				511659	0.8	[59]
			Inhibitory activity against HCV NS3 protease by ELISA	15945060	0.75	[46]

**Table 2:** Pathways modulated by the phytocompounds.

Reactome ID	Pathways	Gene Count	Background Gene Count	FDR	Genes within Pathway
HSA-109582	Hemostasis	8	601	1.52E-05	F10, F11, F2, LCK, PLAT, PLAU, PTPN1, SERPINE1
HSA-140837	Intrinsic pathway of fibrin clot formation	3	22	0.00014	F10, F11, F2
HSA-140877	Formation of fibrin clot (clotting cascade)	3	39	0.00051	F10, F11, F2
HSA-162582	Signal transduction	11	2605	0.00051	F2, HSP90AA1, LCK, PDK1, PLAT, PTPN1, PTPN2, RGS4, RGS7, RGS8, SERPINE1
HSA-9006934	Signaling by receptor tyrosine kinases	5	437	0.0019	HSP90AA1, LCK, PLAT, PTPN1, PTPN2
HSA-76002	Platelet activation, signaling, and aggregation	4	256	0.0023	F2, LCK, PTPN1, SERPINE1
HSA-6807004	Negative regulation of MET activity	2	20	0.0038	PTPN1, PTPN2
HSA-140875	Common pathway of fibrin clot formation	2	22	0.0042	F10, F2
HSA-1280215	Cytokine signaling in immune system	5	654	0.0062	GSTO1, HSP90AA1, LCK, PTPN1, PTPN2
HSA-76009	Platelet aggregation (plug formation)	2	37	0.0096	F2, PTPN1
HSA-449147	Signaling by interleukins	4	439	0.0111	GSTO1, HSP90AA1, LCK, PTPN2
HSA-168256	Immune system	7	1925	0.0245	F2, GSTO1, HSP90AA1, LCK, PLAU, PTPN1, PTPN2

FDR, False discovery rate.

**Table 3:** Binding affinity of prioritized phytocompounds with HCV NS3/4A.

Phytocompounds	PubChem CID	BE (kcal/mol)	Total No. of Interactions	No. of Interactions with Active Site Residues	HBI (Amino Acid…Ligand)	Van der Waals, Pi–Alkyl, CH, Pi–Cation, Pi–Sigma, Pi–Pi Stacked, Pi–Pi T-Shaped Interactions
1,2,3,4,6-Pentagalloyl glucose	374874	−7.7	20	20	Gln1041…OH, His1057…OH (2), Asp1081…OH, Ile1132…OH, Ser1139…O-, Ser1139…OH (2), Arg1155…O- (2)	Val1055, His1057 (2), Gly1058, Ile1132, Lys1136, Ala1156 (3), Ala1157
1,3,4,6-Tetra-O-galloyl-β-D-glucose	471531	−7.6	14	14	Gln1041…OH, Asp1081…OH, Ile1132…OH, Ser1139…OH (2), Ser1139…O- (2), Arg1155…OH	Ile1132, Lys1136 (2), Gly1137, Ala1156, Ala1157
1,3,6-Tri-O-galloyl-β-D-glucose	452707	−7.0	8	8	Gly1058…O-, Gly1137…O-, Ser1139….O- (2), Ser1139…OH	His1057, Lys1136, Ala1156
1,6,-di-O-galloyl-D-glucose	91227631	−6.6	6	5	Thr1042…OH, Ile1132…OH, Ser1139…O-	His1057 (2), Lys1136
2,3,4,6 tetra-O-galloyl-β-D-glucose	101011018	−6.5	11	11	Gln1041…OH, Ser1139…OH (3)	Ile1132, Lys1136, Gly1137, Ser1139, Ala1156 (2), Ala1157
3,4,6-tri-O-galloyl-D-glucose	14188641	−6.6	11	10	Gln1041…OH, Thr1042…OH, Ile1132…OH, Ser1139…OH, Ser1139…O-	His1057, Ile1132, Lys1136, Gly1137, Ala1156, Ala1157
Chebulagic acid	250397	−8.4	13	11	Gly1137…O-, Arg1155…=O, Arg1155…=O, Gly1058…O-, Ile1132…OH (2), Ser1159…OH (2)	Lys1136 (2), His1057, Ile1132, Ser1139
Corilagin	73568	−7.3	3	3	Gln1041…OH, His1057…O-	Lys1136
Tellimagrandin I	442690	−7.7	10	9	Gln1041…OH, Thr1042…OH, Gly1058…O-, Ser1139…=O, Ala1157…O-	His1057, Ile1132 (2), Lys1136, Ala1157
Tercatain	14411426	−7.5	8	6	Leu1135…OH, Ser1139…O-, Ser1159…OH, Ser1159…O-	His1057 (2), Lys1136, Ala1156
* Asunaprevir	16076883	−7.4	20	20	Gly1137…=O (2), Ala1157…NH, Ala1157…=O	Arg1155 (2), Asp1081 (2), His1057 (2), Ala1156 (4), Ile1132 (3), Val1158 (2), Lys1136 (2)

* Standard molecule (HCV NS3/4A inhibitor); BE, binding energy; HBI, hydrogen bond interactions; NHBI, non-hydrogen bond interactions. The interaction of compounds with catalytic triad residues was highlighted.

**Table 4:** Interactions of compounds with HCV NS3/4A after 50ns MD simulation.

Compound/Ligand Name	Amino Acid Residue	Ligand Atom	Ligand Atom Position	Ligand Atom Interactions with the Protein Residues (%)
Asunaprevir	Gly1137	=O	16	97
	Ala1157	=O	7	99
	Ala1157	NH	44	82
	Ser1139	=O	9	56
	Ser1159	-O	47	46
	Gln1041	+O	18	39
	Thr1042	-O	18	38
	Thr1042	-O	18	28
Chebulagic acid	Ala1157	=O	41	61
	Asp1081	OH	68	51
	Asp1081	=O	67	38
	Asp1168	=O	20	31
	Ile1132	OH	46	45
	Arg1155	OH	50	31
	His1057	π-π	25–30	46
1,2,3,4,6-Pentagalloyl glucose	Asp1081	OH	41	99
	Asp1081	OH	40	99
	Arg1155	OH	42	37
	Arg1155	OH	42	22
	Gly1137	OH	65	41
	Ser1139	OH	65	61
	Ser1139	OH	66	72
	Leu1135	OH	67	47
	Leu1157	OH	67	32
	Tyr1056	OH	18	23
	His1057	OH	18	23

**Table 5:** Druglikeness characteristics of phytocompounds.

Phytocompounds	MW (g/mol)	MF	HBA	HBD	LogP	DLS
1,2,3,4,6-Pentagalloyl glucose	940.12	C_41_ H_32_ O_26_	26	15	1.47	0.19
1,3,4,6-Tetra-O-galloyl-β-D-glucose	788.11	C_34_ H_28_ O_22_	22	13	0.62	0.92
1,3,6-Tri-O-galloyl-β-D-glucose	636.1	C_27_ H_24_ O_18_	18	11	−0.08	0.92
1,6,-di-O-galloyl-D-glucose	484.09	C_20_ H_20_ O_14_	14	9	0.8	0.9
2,3,4,6 tetra-O-galloyl-β-D-glucose	788.11	C_34_ H_28_ O_22_	22	13	0.1	0.44
3,4,6-tri-O-galloyl-D-glucose	636.1	C_27_ H_24_ O_18_	18	11	−0.2	0.87
Chebulagic acid	954.1	C_41_ H_30_ O_27_	27	13	0.22	0.58
Corilagin	634.08	C_27_ H_22_ O_18_	18	11	0.51	0.64
Tellimagrandin I	786.09	C_34_ H_26_ O_22_	22	13	1.15	0.3
Tercatain	786.09	C_34_ H_26_ O_22_	22	13	1.21	0.65

MW, molecular weight; MF, molecular formula; HBA, hydrogen bond acceptors; HBD, hydrogen bond donor; DLS, druglikeness score.

## Data Availability

The authors confirm that the data supporting the findings of this study are available within the article (and/or) its Supplementary Materials.

## Supplementary Materials

The following are available online, Table S1: List of phytocompounds from Terminalia chebula; Table S2: Reported pathways involved in the pathogenesis of HCV infection and immune system modulation; Table S3: Probable protein targets modulated by the phytocompounds; Table S4: Phytocompounds predicted to target HCV NS3/4A and other protein molecules involved in immune system; Table S5: Pathways modulated by the protein molecules targeted by the phytocompounds; Table S6: Probability score of phytocompound to exert side effects.
